# Bevacizumab plus chemotherapy for patients with advanced pulmonary adenocarcinoma harboring *EGFR* mutations

**DOI:** 10.1007/s12094-017-1714-2

**Published:** 2017-07-12

**Authors:** R.-L. Chen, H.-J. Chen, B.-Y. Jiang, X.-C. Zhang, Q. Zhou, H.-Y. Tu, W.-Z. Zhong, Y.-L. Wu, J.-J. Yang

**Affiliations:** 1 Guangdong Cardiovascular Institute, Guangdong General Hospital and Guangdong Academy of Medical Sciences, Guangzhou, People’s Republic of China; 2 Guangdong Lung Cancer Institute, Guangdong General Hospital and Guangdong Academy of Medical Sciences, Guangzhou, People’s Republic of China; 3 Medical Research Center, Guangdong General Hospital and Guangdong Academy of Medical Sciences, Guangzhou, People’s Republic of China

**Keywords:** Bevacizumab, *EGFR* mutations, Chemotherapy, EGFR-TKIs

## Abstract

**Purpose:**

Epidermal growth factor receptor (EGFR) tyrosine kinase inhibitors (TKIs) and bevacizumab plus chemotherapy were effective for *EGFR*-mutant patients. However, the appropriated treatment orders remained controvertible. We investigated the efficacy of treatment orders between bevacizumab plus chemotherapy and EGFR-TKIs for *EGFR*-mutant patients with advanced pulmonary adenocarcinoma.

**Patients and methods:**

This study involved 40 *EGFR*-mutant patients with advanced pulmonary adenocarcinoma who were treated with bevacizumab plus carboplatin and paclitaxel (Bev + CP) and EGFR-TKIs in different treatment orders or gemcitabine plus cisplatin (GP) in first-line setting. Seventeen patients were treated with Bev + CP and 10 cases with GP in first-line treatment. Thirteen patients received EGFR-TKIs after first-line Bev + CP regimen, while 13 patients were treated with first-line EGFR-TKIs. Progression-free survival (PFS), the response rate (ORR) and overall survival (OS) were evaluated.

**Results:**

Median PFS of Bev + CP treatment was significantly longer in first-line than non-first-line settings (11.7 vs. 5.6 months, *P* = 0.003). Median OS was 37.8 months for *EGFR*-mutant patients with first-line Bev + CP followed by second-line EGFR-TKIs and 31.0 months for those with first-line EGFR-TKIs and non-first-line Bev + CP, respectively (*P* = 0.509). Median PFS was 11.7 (95% CI 10.6–12.8) months for Bev + CP group and 4.7 (95% CI 4.4–5.0) months for GP group with the hazard ratio of 0.17 (*P* = 0.001). ORR was 70.6 and 50.0% in the two groups, respectively (*P* = 0.415). However, there was no significant difference in median OS (33.7 vs 27.8 months, *P* = 0.293).

**Conclusions:**

First-line Bev + CP followed by EGFR-TKIs might possibly provide favorable prognosis for *EGFR*-mutant patients. Bev + CP regimen significantly prolonged PFS in first-line than non-first-line settings. These findings warrant further investigations.

**Electronic supplementary material:**

The online version of this article (doi:10.1007/s12094-017-1714-2) contains supplementary material, which is available to authorized users.

## Introduction

Lung cancer is a leading cause of death both worldwide and in China [1]. More than 70% of patients present with advanced non-small-cell lung cancer (NSCLC) at the time of diagnosis with poor prognosis. The discovery of driver genes has changed treatment for patients with NSCLC and epidermal growth factor receptor (*EGFR*) is the common driver gene [2–4].

EGFR-TKIs have significantly improved PFS compared with chemotherapy in phase III studies, establishing the utility of their first-line treatment [2, 5–8]. However, there was no significant difference in OS reported. Moreover, chemotherapy is usually used as the subsequent treatment after EGFR TKI failure, resulting the median PFS of only about 4 months [9, 10]. It was also reported that frontline EGFR-TKIs can reduced the sensitivity of subsequent platinum-based doublet chemotherapy [9]. However, the clinical benefit of EGFR-TKIs in first-line treatment was reported to be similar to second-line [11]. It is yet unclear as to what the best sequence of treatment between EGFR-TKIs and chemotherapy. *EGFR*-mutant patients accounted for approximately 30% of NSCLC patients in East Asia, which is more than those of 10% in non-Asia [12, 13]. So, it is vital to figure out the appropriate sequence of treatment administration between EGFR-TKIs and chemotherapy for *EGFR*-mutant NSCLC patients in Asia.

Bevacizumab is a recombinant, humanized monoclonal antibody directed against vascular endothelial growth factor [14]. The bevacizumab plus chemotherapy group showed significantly improvement in both median PFS and OS than the carboplatin and paclitaxel group in several clinical trials [15–17]. A phase II study has demonstrated the median PFS of second-line Bev + CP in *EGFR*-mutant NSCLC patients was 6.6 months, which was shorter than that of first-line Bev + CP with 9.2 months reported in BEYOND trials [15, 18]. So, it is interesting to explore the response of different treatment sequence of EGFR-TKIs and chemotherapy plus bevacizumab in *EGFR*-mutant patients. However, in clinical practice, most patients with *EGFR* mutations received EGFR-TKIs as first-line treatment. Thus, few EGFR-mutant patients received first-line chemotherapy plus bevacizumab, except those enrolled in some clinical trials. Moreover, no study has been reported the efficacy of first-line Bev + CP and second-line of EGFR-TKIs in *EGFR*-mutant patients. Therefore, it inspired us to conduct this retrospective study with most patients enrolled in clinical trials, aiming to investigate OS for *EGFR*-mutant patients according to different treatment orders with respect to Bev + CP and EGFR-TKIs. In addition, only 23 *EGFR*-mutant patients in BEYOND trial were reported to experience prolonged PFS in Bev + CP group compared with CP group [15]. However, there were no data enough to evidence clinical improvement of first-line bevacizumab plus chemotherapy in *EGFR*-mutant patients. Thus, we also retrospectively investigated the efficacy of chemotherapy containing bevacizumab versus chemotherapy alone as first-line treatment in *EGFR*-mutant patients in our institute.

## Patients and methods

### Patients

From August 2006 to January 2016, a total of 40 patients with advanced pulmonary adenocarcinoma harboring *EGFR* mutations were included in the retrospective study at the Guangdong Lung Cancer Institute (GLCI). The inclusion criteria were (1) pathologically confirmed advanced pulmonary adenocarcinoma with at least one measurable lesion and an ECOG performance status of 0–2; (2) identified with *EGFR* mutations; (3) received GP or Bev + CP regimen as first-line treatment in clinical trials or received EGFR-TKIs as a first-line treatment followed by Bev + CP regimen in the second- or further-line settings. Patients were categorized as those who had never smoked (<100 lifetime cigarettes) and smokers who had a history of smoking more than 100 cigarettes lifetime. The patients were identified using electronic medical and radiographic records at GLCI and all tissues used for this study were from the GLCI tissue bank. This study was approved by the Institutional Review Board of Guangdong General Hospital (GGH, Guangzhou, China) and informed consent for molecular analyses was obtained from each patient.

### Study design and treatment

**Fig. 1:**
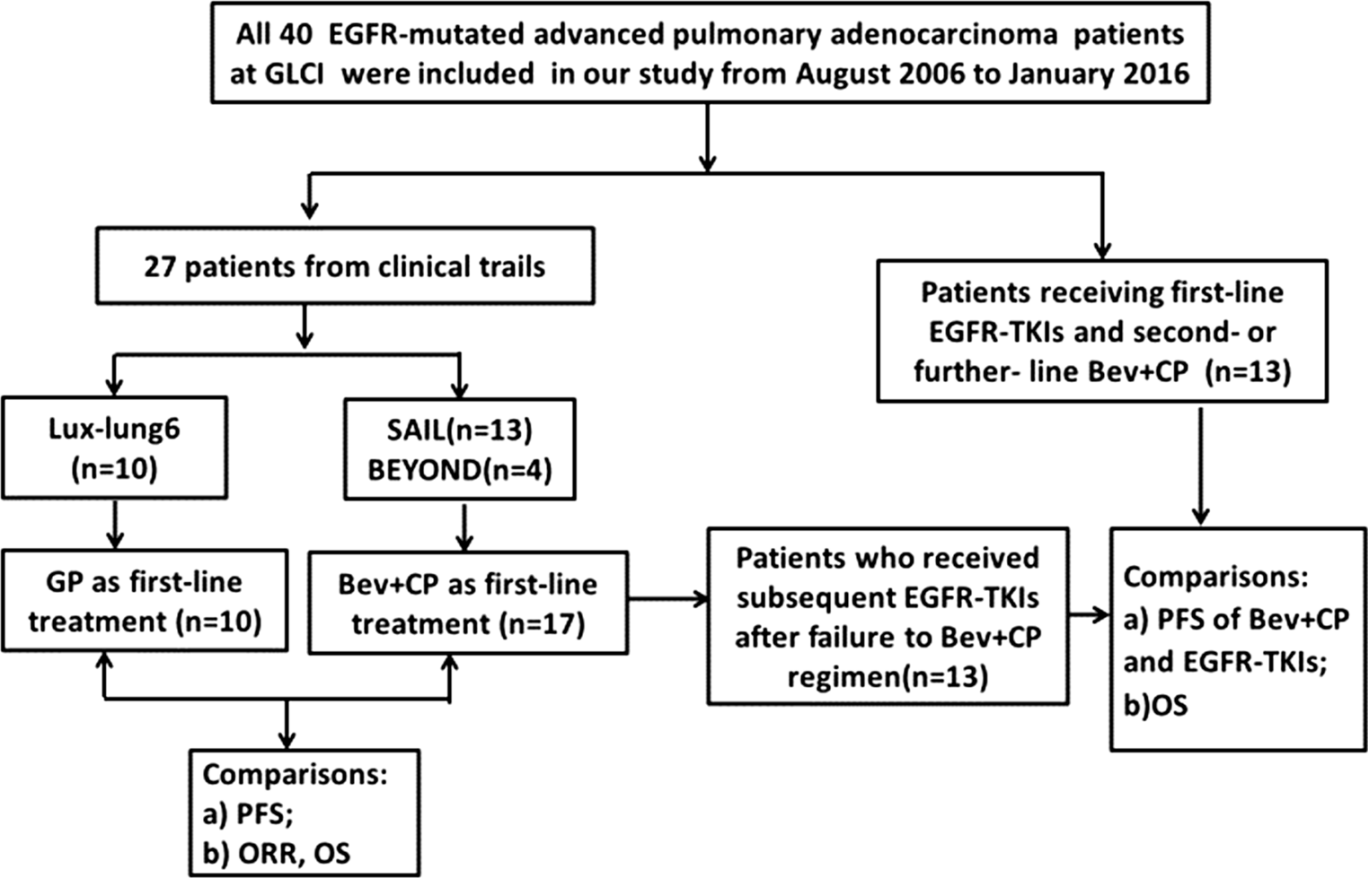
Study flow chart. *Bev* *+* *CP* bevacizumab plus carboplatin and paclitaxel, *ORR* objective response rate, *PFS* progression-free survival, *OS* overall survival, *GP* gemcitabine and cisplatin, *GLCI* Guangdong Lung Cancer Institute

Our study enrolled 40 *EGFR*-mutant patients (Fig. 1). Importantly, according to the different treatment orders, we divided patients into two groups: one group included 13 *EGFR*-mutant patients that received first-line Bev + CP regimen followed by EGFR-TKIs; the other group consisted of 13 cases treated with first-line EGFR-TKIs and second- or further-line Bev + CP regimen. In addition, to explore the efficacy of Bev + CP treatment for *EGFR*-mutant patients, the present study compared 17 cases that received Bev + CP treatment as first-line setting with 10 patients treated with GP regimen.

Seventeen *EGFR*-mutant patients from BEYOND and SAiL trials in Bev + CP group received bevacizumab plus carboplatin and paclitaxel (bevacizumab, 15 mg/kg, carboplatin, area under the curve = 6, paclitaxel, 175 mg/m^2^) every 3 weeks for up to six cycles and maintaining with bevacizumab as first-line treatment until disease progression, intolerable toxicity, or patient withdrawal [15, 16]. Of whom 13 *EGFR*-mutant patients received EGFR-TKIs as second-line after failure to treatment of Bev + CP (6 patients with gefitinib at a dose of 250 mg daily, 7 with erlotinib at a dose of 150 mg daily). In addition, 13 *EGFR*-mutant patients were treated with Bev + CP regimen as second- or further-line after first-line EGFR-TKIs (8 with gefitinib at a dose of 250 mg daily, 3 with erlotinib at a dose of 150 mg daily, 1 with afatinib at a dose of 40 mg daily and 1 with icotinib at a dose of 125 mg three time a day). Ten *EGFR*-mutant patients who enrolled in Lux-Lung 6 trial received intravenous gemcitabine (1000 mg/m^2^, on day 1 and day 8) plus cisplatin (75 mg/m^2^, on day 1) in a 3-week schedule until disease progression, intolerable toxic effects, or withdrawal of consent. Gemcitabine and cisplatin was given for a maximum of six cycles [19].

### *EGFR* mutations analysis

Tumor histology was classified using the World Health Organization criteria. All the tumor samples were conducted by two independent pathologists to ensure that the specimen contained at least 50% cancer cells before *EGFR* analysis. *EGFR* mutations were identified by any of the two methods: direct DNA sequencing or the Scorpion Amplification Refractory Mutation System (ARMS) method.

### Evaluation of efficacy

Tumors of 10 patients in Lux-Lung 6 trial were assessed by CT scan every 6 weeks for the first 48 weeks, then subsequently every 12 weeks until objective disease progression or start of further cancer treatment [20]. For 4 patients in BEYOND trial, tumor response was assessed at the end of every second treatment cycle or 6 weeks until progression according to the response evaluation criteria in solid tumors (RECIST) [21]. Tumor assessments of 13 patients from SAiL trial were based on the treating physicians’ clinical practice [16]. The evaluation of tumor response for other patients at the GLCI was assessed by CT scan every 6–8 weeks according to RECIST [21]. ORR was defined as the best tumor response in cases with complete response (CR) and partial response (PR) that was confirmed and sustained for at least eight weeks. PFS was defined as the period from the start of treatment to the date when disease progression or death was observed. OS was defined as the period from the date of diagnosis of locally advanced or metastatic (stage IV) to the date of death. Toxicity was graded according to the United States National Cancer Institute’s common toxicity criteria version 4.0.

### Statistical analyses

The survival distributions (PFS and OS) were estimated by using the Kaplan–Meier method, and differences among subgroups were compared using the log-rank test. Comparison of ORRs and the baseline clinical characteristics in different groups were performed using *X* ^2^ tests. Statistical analysis was performed using SPSS version 22.0 software (IBM, Armonk, NY).

## Results

### Patient populations

From August 2006 to January 2016, a total of 40 treatment-naive *EGFR*-mutant patients with advanced pulmonary adenocarcinoma were retrospectively evaluated at the GLCI. 17 patients received bevacizumab plus paclitaxel and carboplatin for up to six cycles and maintaining with bevacizumab, while 10 were treated with gemcitabine and cisplatin. The median age was 57 years (range 40–70) in bevacizumab plus chemotherapy group and 53 years (range 30–65) in chemotherapy alone group, with the majority of patients in both groups having Eastern Cooperative Oncology Group performance status 1, stage IV disease and adenocarcinoma histology (Table 1). More female was found in GP group than in Bev + CP group. Median follow-up time was 33.7 months for Bev + CP group and 27.8 months for GP group.

**Table 1:** Baseline clinical characteristics in *EGFR*-mutated patients with advanced pulmonary adenocarcinoma receiving Bev + CP and GP regimens

Characteristic	No. of patients (%)		*P* value
	Bev + CP (*n* = 17)	GP (*n* = 10)	
Age, years			
Median	57	53	0.815
Range	40–70	30–65	
Gender			
Male	10 (58.8%)	3 (30.0%)	0.236
Female	7 (41.2%)	7 (70.0%)	
ECOG PS			
0–1	17 (100.0%)	9 (90.0%)	0.370
2	0 (0.0%)	1 (0.0%)	
Smoking status			
Nonsmoker	10 (58.8%)	9 (97.4%)	0.190
Smoker	7 (41.2%)	1 (2.6%)	
Clinical staging			
III B	1 (5.9%)	0 (0.0%)	1.000
IV	16 (94.1%)	10 (100.0%)	
*EGFR* mutation status			
19 deletion	10 (58.8%)	8 (80.0%)	0.406
L858R mutation	5 (29.4%)	2 (20.0%)	
Others	2 (11.8%)	0 (0.0%)	

*Bev* *+* *CP* bevacizumab, carboplatin, and paclitaxel; *GP* gemcitabine plus cisplatin; *ECOG PS* Eastern Cooperative Oncology Group performance status; *EGFR* epidermal growth factor receptor

Of 17 *EGFR*-mutant patients failing first-line Bev + CP treatment, 13 cases were treated with EGFR-TKIs as subsequent therapy. Another 13 *EGFR*-mutant patients who received Bev + CP regimen in second- or further-line anti-cancer treatment after failure in first-line EGFR-TKIs were included in our study for comparison. More female and non-smokers were found in first-line Bev + CP followed by EGFR-TKIs group than in first-line EGFR-TKIs and non-first-line Bev + CP group (Supplementary Table S1). Median follow-up time was 33.7 months for first-line Bev + CP followed EGFR-TKIs group and 29.8 months for first-line EGFR-TKIs and non-first-line Bev + CP group.

### The efficacy of bevacizumab plus chemotherapy in patients with *EGFR* mutations

**Fig. 2:**
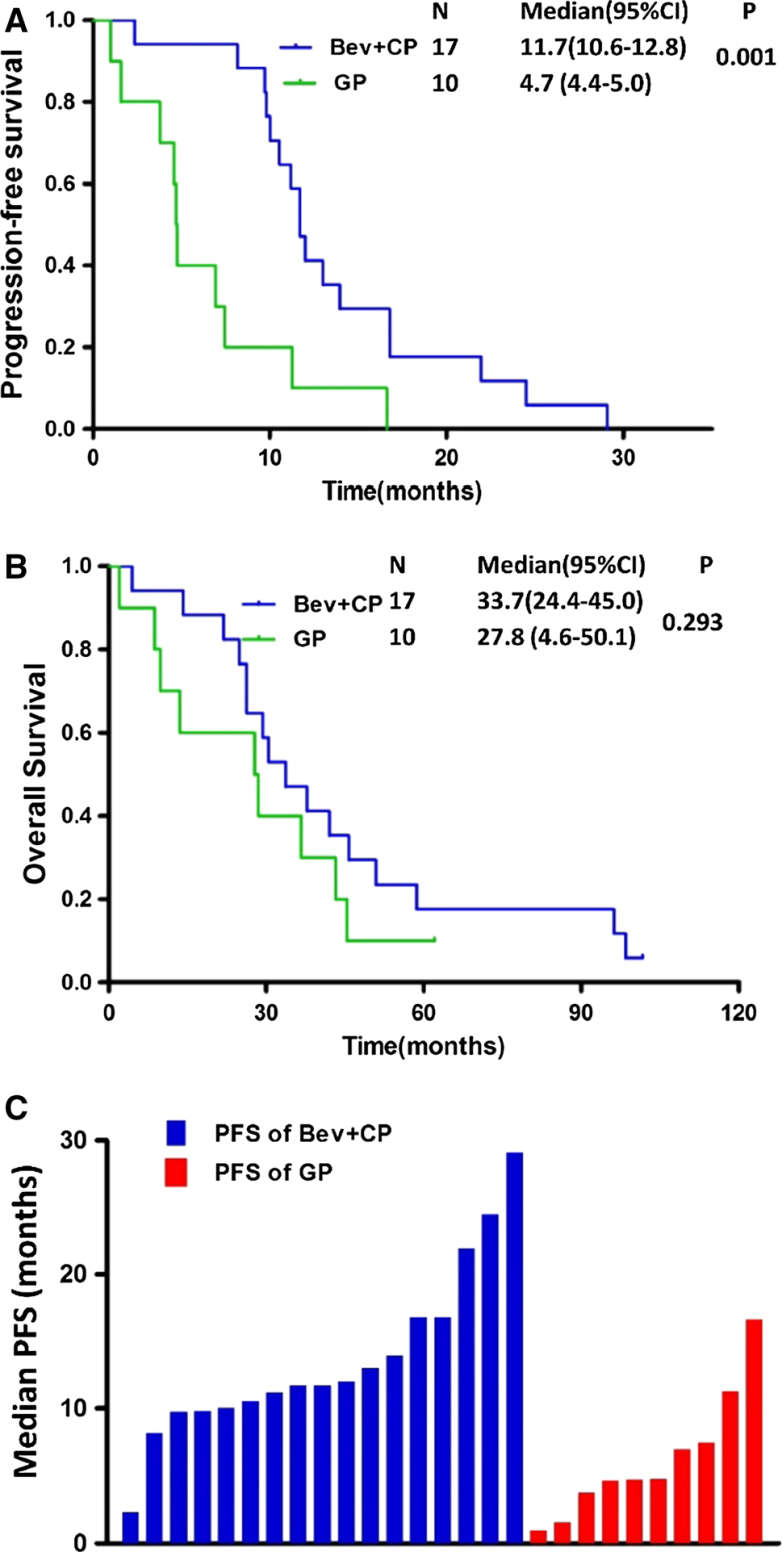
Kaplan–Meier curve of progression-free survival (**a**) and overall survival (**b**) in patients harboring *EGFR* with *E*GFR mutation. Median progression-free survival of all patients in Bev + CP and GP groups. (**c**) Bev + CP patients treated with bevacizumab plus chemotherapy with carboplatin and paclitaxel, GP patients treated with gemcitabine and cisplatin regimen

A total of 27 *EGFR*-mutant patients (17 in bevacizumab plus chemotherapy and 10 in chemotherapy alone group) were assessed. The median PFS was 11.7 months for *EGFR*-mutant patients in bevacizumab plus chemotherapy group and 4.7 months for those in GP group, with significant difference (HR = 0.16, 95% CI 0.06–0.50, *P* = 0.001) (Fig. 2a–c). There was no significant difference in median OS for the patients with *EGFR* mutations, 33.7 months in Bev + CP and 27.8 months in GP group (HR = 0.61, 95% CI 0.25–1.52 *P* = 0.293) (Fig. 2b). Objective response rate (ORR) was 70.6% (12/17) in *EGFR*-mutant NSCLC patients treated with bevacizumab plus chemotherapy and 50.0% (5/10) in those with chemotherapy alone (*P* = 0.415) (Table 2).

**Table 2:** ORRs of bevacizumab plus CP versus GP regimen

Response	No. of patients (%)		*P* value
	Bev + CP (*n* = 17)	GP (*n* = 10)	
Partial response, *n* (%)	12 (70.6%)	5 (50.0%)	
Stable disease, *n* (%)	4 (23.5%)	5 (50.0%)	
Progressive disease, *n* (%)	1 (5.9%)	0 (0%)	
ORR, %	70.6%	50.0%	0.415

*Bev* *+* *CP* bevacizumab plus carboplatin and paclitaxel, *ORR* objective response rate, *GP* gemcitabine and cisplatin regimen

### Efficacy of different treatment orders between EGFR-TKIs and Bev + CP regimens in *EGFR*-mutant patients

**Fig. 3:**
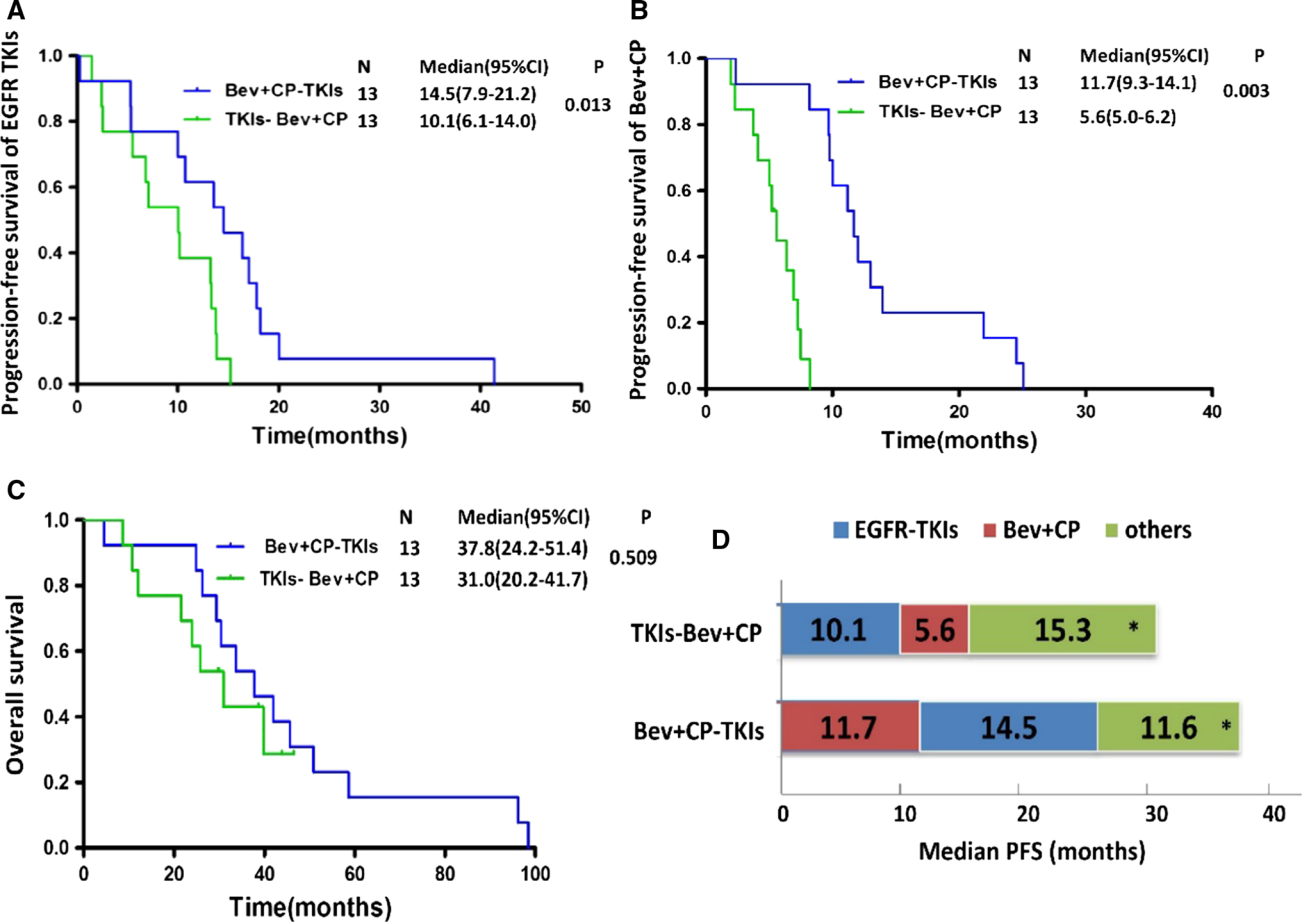
Progression-free survival of EGFR-TKIs (**a**) and Bev + CP treatment (**b**) in Bev + CP-TKIs and TKIs-Bev + CP groups; Kaplan–Meier curve of overall survival (**c**) in two groups; clinical course of *EGFR*-mutant patients receiving first-line Bev + CP or second- or further-line Bev + CP (**d**) Bev + CP-TKIs, patients treated with first-line Bev + CP and second-line EGFR-TKIs; TKIs- Bev + CP, patients treated with first-line EGFR-TKIs and non-first-line Bev + CP; (*asterisk*) calculated by OS and PFS

The median PFS of Bev + CP regimen in first-line therapy was 11.7 months (95% CI 9.3–14.1), compared with 5.6 months (95% CI 5.0–6.2) in the non-first-line settings (HR = 0.07, 0.02227–0.2133, *P* = 0.003) (Fig. 3b). Patients treated with EGFR-TKIs as the first-line experienced a median PFS of 14.5 months (95% CI 7.9–21.2) compared with 10.1 months (95% CI 6.1–14.0) in those who received EGFR-TKIs after failure to Bev + CP regimen (HR = 0.31, 0.1220–0.7784, *P* = 0.013) (Fig. 3a). The median OS was 37.8 months for *EGFR*-mutant patients with first-line Bev + CP followed by second-line EGFR-TKIs, numerically better than 31.0 months for those with first-line EGFR-TKIs followed by second- or further-line Bev + CP regimen, but with no significant difference (HR = 0.72, 0.2664–1.927, *P* = 0.509) (Fig. 3c, d). All of 26 EGFR-positive patients were available for response evaluation of bevacizumab plus chemotherapy. There was no statistically significant difference in the ORR of Bev + CP in first-line regimen compared with that in non-first-line treatment (61.2 vs 69.2%, *P* = 1.000).

**Fig. 4:**
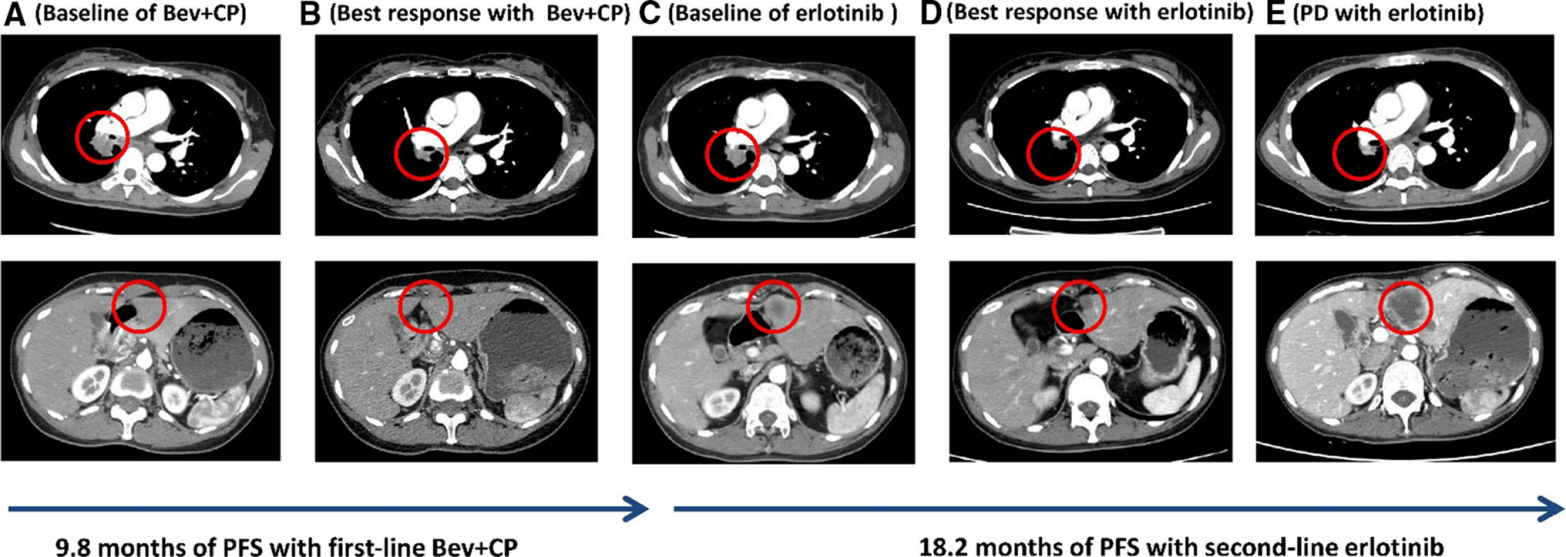
Computed tomography (CT) scans of one of our patients treated with first-line CP regimen plus bevacizumab followed by second-line EGFR-TKIs. **a** Chest CT scans before CP plus bevacizumab. **b** Partial response after 4 months with CP plus bevacizumab. **c** Disease progression after 9.8 months of CP plus bevacizumab treatment. **d** Partial response after 2 months with eroltinib. **e** Disease progression after 18.2 months of erlotinib treatment

A typical case with advanced pulmonary adenocarcinoma harboring *EGFR* 19 deletion experienced PR and long duration of response treated with first-line Bev + CP and second-line erlotinib (Fig. 4). The patients obtained PFS of 9.8 months with first-line Bev + CP and 18.2 months with second-line erlotinib.

### Adverse events

Main toxicities possibly related to therapy were listed in Table 3. The most common adverse events (incidence ≥30%, any grade) of chemotherapeutical regimens were hematologic toxicities. Grade 3–4 adverse events included neutropenia (4/17, 23.5%); proteinuria (1/17, 5.9%) and anemia (1/17, 5.9%) in first-line Bev + CP treatment; leukopenia (1/10, 10.0%); neutropenia (1/10, 10.0%); anemia (1/10, 10.0%) and thrombocytopenia (1/10, 10.0%) in first-line GP treatment, neutropenia (3/13, 23.1%) and anemia (1/13, 7.7%) in further-line Bev + CP treatment. Adverse events of EGFR-TKIs in first-or second-line were generally mild, ranging from grade 1 to grade 2, mainly including rash and diarrhea. No patients experienced drug-related deaths in our study.

**Table 3:** Adverse events of chemotherapy plus bevacizumab, chemotherapy alone and EGFR-TKIs in *EGFR*-mutant NSCLC

Grade *n* (%)	First-line Bev + CP (*n* = 17)				First-line GP (*n* = 10)				Further-line Bev + CP (*n* = 13)				First-line EGFR-TKIs (*n* = 13)				Second-line EGFR-TKIs (*n* = 13)			
	1	2	3	4	1	2	3	4	1	2	3	4	1	2	3	4	1	2	3	4
Leukopenia	1 (5.9)	2 (11.8)	0	0	0	0	1 (10.0)	0	2 (15.4)	1 (7.7)	0	0	0	0	0	0	0	0	0	0
Neutropenia	0	2 (11.8)	3 (17.6)	1 (5.9)	0	2 (20.0)	0	1 (10.0)	1 (7.7)	1 (7.7)	2 (15.4)	1 (7.7)	0	0	0	0	0	0	0	0
Anemia	3 (17.6)	2 (11.8)	1 (5.9)	0	1 (10.0)	2 (20.0)	1 (10.0)	0	4 (30.8)	2 (15.4)	1 (7.7)	0	0	0	0	0	0	0	0	0
Thrombocytopenia	1 (5.9)	0	1 (5.9)	0	2 (20.0)	0	1 (10.0)	0	2 (15.4)	1 (7.7)	0	0	0	0	0	0	0	0	0	0
ALT increased	1 (5.9)	0	0	0	1 (10.0)	0	0	0	1 (7.7)	0	0	0	1 (7.7)	0	0	0	1 (7.7)	0	0	0
AST increased	1 (5.9)	0	0	0	1 (10.0)	0	0	0	1 (7.7)	0	0	0	0	0	0	0	1 (7.7)	0	0	0
Proteinuria	1 (5.9)	2 (11.8)	0	1 (5.9)	1 (10.0)	0	0	0	2 (15.4)	1 (7.7)	0	0	0	0	0	0	0	0	0	0
Bleeding	1 (5.9)	0	0	0	1 (10.0)	0	0	0	1 (7.7)	0	0	0	0	0	0	0	0	0	0	0
Hypertension	3 (17.6)	1 (5.9)	0	0	0	0	0	0	5 (38.5)	1	0	0	0	0	0	0	0	0	0	0
Rash	1 (5.9)	0	0	0	0	0	0	0	1 (7.7)	0	0	0	7 (53.8)	2 (15.4)	0	0	6 (46.2)	2 (15.4)	0	0
Diarrhea	1 (5.9)	0	0	0	1 (10.0)	0	0	0	0	1 (7.7)	0	0	5 (38.5)	2 (15.4)	0	0	6 (46.2)	2 (15.4)	0	0
Fatigue	2 (11.8)	2 (11.8)	0	0	1 (10.0)	1 (10.0)	0	0	3 (23.1)	1 (7.7)	0	0	1 (7.7)	0	0	0	1 (7.7)	1 (7.7)	0	0
Nausea	1 (5.9)	0	0	0	2 (20.0)	1 (10.0)	0	0	1 (7.7)	0	0	0	1 (7.7)	0	0	0	1 (7.7)	0	0	0
Vomiting	2 (11.8)	0	0	0	3 (30.0)	1 (10.0)	0	0	2 (15.4)	1 (7.7)	0	0	0	0	0	0	1 (7.7)	0	0	0

*AE* adverse events, *Bev* *+* *CP* bevacizumab plus carboplatin and paclitaxel, *GP* gemcitabine and cisplatin regimen

## Discussion

Our analysis showed that the addition of bevacizumab to CP provided a meaningful benefit in terms of PFS in *EGFR*-mutant patients. Importantly, our study indicated that a prolonged PFS of Bev + CP regimen was obtained in first-line setting than second- or further-line one in *EGFR*-mutant patients. Meanwhile, it seemed that a trend of improvement of median OS was observed in *EGFR*-mutant patients with first-line Bev + CP followed by second-line EGFR-TKIs.

The ECOG 1594 study demonstrated that gemcitabine plus cisplatin as first-line treatment for metastatic NSCLC results in a small but statistically significant improvement on time to progression of disease, as compared with paclitaxel plus carboplatin (4.2 vs. 3.1 months) [22]. Thus, our study conducted gemcitabine plus cisplatin for comparison with Bev + CP. In a randomized phase III trial (ECOG 4599) among Western population, median PFS was just 6.2 months in the bevacizumab-contained treatment as first-line setting [17]. Besides, median TTP was 7.8 months in bevacizumab-based arm in SAiL study [16]. BEYOND trial demonstrated median PFS of 9.2 months of Bev + CP treatment in treat-naïve NSCLC patients, as compared with 6.5 months of chemotherapy alone [15]. Our study confirmed that first-line bevacizumab plus chemotherapy obtained a significant improvement of PFS compared with GP regimen in patients with *EGFR* mutations, which was consistent with the subgroup analysis of BEYOND in 23 *EGFR*-mutant patients with the median PFS of 12.4 months (Supplementary Table S2).

Median OS in ECOG 4599 and SAiL trials were 12.3 and 14.6 months for patients with bevacizumab plus chemotherapy, respectively [16, 17]. Median OS of BEYOND trial in *EGFR*-mutant patients was 24.3 and 27.5 months for bevacizumab plus CP group and placebo plus CP regimen, respectively [15]. However, our study demonstrated median OS was 33.7 months for *EGFR*-mutant patients with chemotherapy containing bevacizumab, numerically better than those of 27.8 months with chemotherapy with HR of 0.90, which is inconsistent with the sub-analysis of *EGFR*-mutant patients in BEYOND trail [15]. It may be due to most of patients in bevacizumab plus chemotherapy group with EGFR-TKIs as subsequent anti-tumor therapy in our study. Approximately 76.5% (13/17) of *EGFR*-mutant patients received EGFR-TKIs after failing bevacizumab plus chemotherapy, while only 23 *EGFR*-mutant cases in Bev + CP group and about 36% of this group were treated with EGFR-TKIs as subsequent line in BEYOND trials. However, the median PFS and median OS in our study for the first-line bevacizumab plus chemotherapy (PFS median 11.7 months, HR 0.17, OS median 33.7 months, HR 0.61) was longer than that in several clinical trials previously reported [16, 17] (Supplementary Table S2), which mainly contributed to all patients with *EGFR* mutations. It was indicated that the status of *EGFR* may be associated with the improvement of PFS. There was no significant difference in ORR and OS between Bev + CP and GP groups, which may mostly be attributable to the small size sample in our study.

EGFR-TKIs have been considered as first-line treatment for encouraging improvement on PFS based on several clinical trials [2, 5–8]. However, bevacizumab was not given in combination with cytotoxic agents in any studies as comparison. Thus, the efficacy of EGFR-TKIs versus chemotherapy containing bevacizumab is still under exploration. A retrospective study demonstrated the median PFS (11.0 vs. 10.2 months) of EGFR-TKIs was similar between first- and second-line treatments [9]. The median PFS and response rate of EGFR-TKI were reported to have no meaningful difference if given as first- or second- line in a phase II study [11]. However, our study showed that median PFS of EGFR-TKIs was prolonged in second-line than in first-line setting, which may partially be attributable to different influence of first-line treatment on subsequent anti-cancer therapy. It predicted that bevacizumab in the former-line treatment simply potentiates the effect of later-line of EGFR-TKIs on both normal and malignant cells. Our data further confirmed that frontline chemotherapy could not reduce efficacy of subsequent EGFR-TKIs in *EGFR*-mutant patients reported in previous study [9]. Recently, in large sample-size clinical trials, first-line bevacizumab plus chemotherapy has been evidenced to provide clinical benefit in patients with or without *EGFR* mutations [15, 16, 23]. However, different orders of anti-cancer treatments may make impact on survival benefit in patients with pulmonary adenocarcinoma. A retrospective analysis demonstrated that bevacizumab plus chemotherapy encouraged anti-tumor efficacy as both first- and second-line therapy in advanced patients with lung cancer [24]. The TORCH randomized clinical trial demonstrated the response rate of chemotherapy was not influenced by first-line treatment of EGFR-TKIs in unselected Caucasian population [25]. According to NEJ002 study, prior EGFR-TKIs therapy would not influence the efficacy of subsequent chemotherapy in patients harboring *EGFR* mutations. However, patients with first-line EGFR-TKIs were reported to be less sensitive to subsequent chemotherapy than those with first-line chemotherapy [9]. Thus, it may be an issue which is the appropriate medication orders between bevacizumab plus chemotherapy and EGFR-TKIs for patients harboring *EGFR* mutations.

Our study firstly demonstrated the clinical efficacy of different treatment orders between EGFR-TKIs and bevacizumab plus chemotherapy in *EGFR*-mutant patients. Median OS in patients treated with first-line Bev + CP and second-line EGFR-TKIs was 37.8 months, numerically better than 31.0 months for those with first-line EGFR-TKIs and non-first-line Bev + CP, although there was no significant difference. Safety analysis of our study also showed a generally low incidence of grade 3 or more AEs of first-line Bev + CP. Our analysis revealed that an improvement of PFS in Bev + CP regimen was obtained in first-line setting than non-first-lines in *EGFR*-mutant patients (11.7 vs 5.6 months, *P* = 0.003). A phase II study from Japanese researchers demonstrated that median PFS of 6.6 months and median OS of 18.2 months for 30 *EGFR*-mutant patients with second-line Bev + CP after failure to first-line EGFR-TKIs [26]. However, in BEYOND trial, *EGFR*-mutant patients received first-line Bev + CP obtained median PFS of 12.4 months and median OS of 24.3 months [15]. Furthermore, our results demonstrated that the PFS of EGFR-TKIs in second-line was significantly prolonged than in first-line. It predicted that first-line bevacizumab may improve sensitivity to subsequent EGFR-TKIs in *EGFR*-mutant patients. These findings and our data indicated that bevacizumab plus chemotherapy as first-line treatment might provide more clinical benefit on PFS and OS than as non-first-line therapy in *EGFR*-mutant patients. Thus, pulmonary adenocarcinoma patients harboring *EGFR* mutations may achieve better response with first-line bevacizumab plus chemotherapy followed by second-line EGFR-TKIs. However, because of the small sample-size in our study and the previous limited evidence, more studies will be needed to confirm this strategy of treatment.

Although the majority of patients in our study were from clinical trials, there were several limitations. First, this was a single-center analysis with non-synchronous trial and unmatched populations. Second, baseline characteristics were not well balanced between two arms for comparison. The history of smoking was poor prognosis of survival for *EGFR*-mutant patients [27, 28]. Although more smokers in patients with first-line Bev + CP followed by EGFR-TKIs, a trend of improvement on prognosis was still seen in this group. Furthermore, we could not collect the data of patients’ QoL in present study. Finally, our study only included a small sample size of patients.

In conclusion, bevacizumab plus chemotherapy might possibly favor *EGFR*-mutant patients, compared with chemotherapy alone. For patients with *EGFR* mutations, bevacizumab plus platinum-based doublet chemotherapy as first-line treatment might provide favorable prognosis than as second- or further-line regimen. This treatment strategy warrants a large number of further investigations to be valid.

## Electronic supplementary material

Below is the link to the electronic supplementary material.

Supplementary material 1 (DOCX 17 kb)

## References

[1] Siegel RL, Miller KD, Jemal A (2016). Cancer statistics, 2016. CA Cancer J Clin.

[2] Mok TS, Wu YL, Thongprasert S, Yang CH, Chu DT, Saijo N (2009). Gefitinib or carboplatin-paclitaxel in pulmonary adenocarcinoma. N Engl J Med.

[3] Shaw AT, Kim DW, Nakagawa K, Seto T, Crino L, Ahn MJ (2013). Crizotinib versus chemotherapy in advanced ALK-positive lung cancer. N Engl J Med.

[4] Fukuoka M, Wu YL, Thongprasert S, Sunpaweravong P, Leong SS, Sriuranpong V (2011). Biomarker analyses and final overall survival results from a phase III, randomized, open-label, first-line study of gefitinib versus carboplatin/paclitaxel in clinically selected patients with advanced non-small-cell lung cancer in Asia (IPASS). J Clin Oncol.

[5] Maemondo M, Inoue A, Kobayashi K, Sugawara S, Oizumi S, Isobe H (2010). Gefitinib or chemotherapy for non-small-cell lung cancer with mutated EGFR. N Engl J Med.

[6] Mitsudomi T, Morita S, Yatabe Y, Negoro S, Okamoto I, Tsurutani J (2010). Gefitinib versus cisplatin plus docetaxel in patients with non-small-cell lung cancer harbouring mutations of the epidermal growth factor receptor (WJTOG3405): an open label, randomised phase 3 trial. Lancet Oncol.

[7] Zhou C, Wu YL, Chen G, Feng J, Liu XQ, Wang C (2011). Erlotinib versus chemotherapy as first-line treatment for patients with advanced EGFR mutation-positive non-small-cell lung cancer (OPTIMAL, CTONG-0802): a multicentre, open-label, randomised, phase 3 study. Lancet Oncol.

[8] Rosell R, Carcereny E, Gervais R, Vergnenegre A, Massuti B, Felip E (2012). Erlotinib versus standard chemotherapy as first-line treatment for European patients with advanced EGFR mutation-positive non-small-cell lung cancer (EURTAC): a multicentre, open-label, randomised phase 3 trial. Lancet Oncol.

[9] Zeng Z, Yan H, Zhang X, Zhong W, He Y, Guan J (2014). Reduced chemotherapy sensitivity in EGFR-mutant lung cancer patient with frontline EGFR tyrosine kinase inhibitor. Lung Cancer.

[10] Ettinger DS, Wood DE, Akerley W, Bazhenova LA, Borghaei H, Camidge DR (2016). NCCN guidelines insights: non-small cell lung cancer, version 4.2016. J Natl Compr Cancer Netw.

[11] Rosell R, Moran T, Queralt C, Porta R, Cardenal F, Camps C (2009). Screening for epidermal growth factor receptor mutations in lung cancer. N Engl J Med.

[12] Paez JG, Janne PA, Lee JC, Tracy S, Greulich H, Gabriel S (2004). EGFR mutations in lung cancer: correlation with clinical response to gefitinib therapy. Science.

[13] Sekine I, Yamamoto N, Nishio K, Saijo N (2008). Emerging ethnic differences in lung cancer therapy. Br J Cancer.

[14] Presta LG, Chen H, O’Connor SJ, Chisholm V, Meng YG, Krummen L (1997). Humanization of an anti-vascular endothelial growth factor monoclonal antibody for the therapy of solid tumors and other disorders. Cancer Res.

[15] Zhou C, Wu YL, Chen G, Liu X, Zhu Y, Lu S (2015). BEYOND: a randomized, double-blind, placebo-controlled, multicenter, phase III study of first-line carboplatin/paclitaxel plus bevacizumab or placebo in chinese patients with advanced or recurrent nonsquamous non-small-cell lung cancer. J Clin Oncol.

[16] Crino L, Dansin E, Garrido P, Griesinger F, Laskin J, Pavlakis N (2010). Safety and efficacy of first-line bevacizumab-based therapy in advanced non-squamous non-small-cell lung cancer (SAiL, MO19390): a phase 4 study. Lancet Oncol.

[17] Sandler A, Yi J, Dahlberg S, Kolb MM, Wang L, Hambleton J (2010). Treatment outcomes by tumor histology in Eastern Cooperative Group Study E4599 of bevacizumab with paclitaxel/carboplatin for advanced non-small cell lung cancer. J Thorac Oncol.

[18] Hattori Y, Satouchi M, Shimada T, Urata Y, Yoneda T, Mori M (2015). A phase 2 study of bevacizumab in combination with carboplatin and paclitaxel in patients with non-squamous non-small-cell lung cancer harboring mutations of epidermal growth factor receptor (EGFR) after failing first-line EGFR-tyrosine kinase inhibitors (HANSHIN Oncology Group 0109). Lung Cancer.

[19] Yang JC, Wu YL, Schuler M, Sebastian M, Popat S, Yamamoto N (2015). Afatinib versus cisplatin-based chemotherapy for EGFR mutation-positive lung adenocarcinoma (LUX-Lung 3 and LUX-Lung 6): analysis of overall survival data from two randomised, phase 3 trials. Lancet Oncol.

[20] Wu YL, Zhou C, Hu CP, Feng J, Lu S, Huang Y (2014). Afatinib versus cisplatin plus gemcitabine for first-line treatment of Asian patients with advanced non-small-cell lung cancer harbouring EGFR mutations (LUX-Lung 6): an open-label, randomised phase 3 trial. Lancet Oncol.

[21] Therasse P, Arbuck SG, Eisenhauer EA, Wanders J, Kaplan RS, Rubinstein L (2000). New guidelines to evaluate the response to treatment in solid tumors. European Organization for Research and Treatment of Cancer, National Cancer Institute of the United States, National Cancer Institute of Canada. J Natl Cancer Inst.

[22] Schiller JH, Harrington D, Belani CP, Langer C, Sandler A, Krook J (2002). Comparison of four chemotherapy regimens for advanced non-small-cell lung cancer. N Engl J Med.

[23] Sandler A, Gray R, Perry MC, Brahmer J, Schiller JH, Dowlati A (2006). Paclitaxel-carboplatin alone or with bevacizumab for non-small-cell lung cancer. N Engl J Med.

[24] Quan R, Huang J, Chen N, Fang W, Hu Z, Zhan J (2016). A retrospective analysis of efficacy and safety of adding bevacizumab to chemotherapy as first- and second-line therapy in advanced non-small-cell lung cancer (NSCLC). Tumour Biol.

[25] Gridelli C, Ciardiello F, Gallo C, Feld R, Butts C, Gebbia V (2012). First-Line erlotinib followed by second-line cisplatin-gemcitabine chemotherapy in advanced non-small-cell lung cancer: the torch randomized trial. J Clin Oncol.

[26] Hattori Y, Satouchi M, Shimada T, Urata Y, Yoneda T, Mori M (2015). A phase 2 study of bevacizumab in combination with carboplatin and paclitaxel in patients with non-squamous non-small-cell lung cancer harboring mutations of epidermal growth factor receptor (EGFR) after failing first-line EGFR-tyrosine kinase inhibitors (HANSHIN Oncology Group 0109). Lung Cancer.

[27] Kim MH, Kim HR, Cho BC, Bae MK, Kim EY, Lee CY (2014). Impact of cigarette smoking on response to epidermal growth factor receptor (EGFR)-tyrosine kinase inhibitors in lung adenocarcinoma with activating EGFR mutations. Lung Cancer.

[28] Hasegawa Y, Ando M, Maemondo M, Yamamoto S, Isa SI, Saka H (2015). The role of smoking status on the progression-free survival of non-small cell lung cancer patients harboring activating epidermal growth factor receptor (EGFR) mutations receiving first-line EGFR tyrosine kinase inhibitor versus platinum doublet chemotherapy: a meta-analysis of prospective randomized trials. Oncologist.

