# Supplementary material for: Bevacizumab plus chemotherapy for patients with advanced pulmonary adenocarcinoma harboring EGFR mutations
Source: Clin Transl Oncol. 2017 Jul 12;20(2):243–52. doi: 10.1007/s12094-017-1714-2 (PMC5797192; doi:10.1007/s12094-017-1714-2)
Supplement: Supplementary file 1 — Supplementary material 1 (DOCX 17 kb) [file 12094_2017_1714_MOESM1_ESM.docx]

**Supplementary Materials**

**Table S1. Baseline clinical characteristics in *EGFR*-mutated patients receiving Bev+CP-TKIs and TKIs-Bev+CP regimens.**

| Characteristic | No. of Patients (%) | | P-value |
| --- | --- | --- | --- |
|  | **Bev+CP-TKIs (n=13)** | **TKIs-Bev+CP (n=13)** |  |
| Age, years |  |  |  |
| Median | 55 | 56 | 0.496 |
| Range | 34-73 | 36-76 |  |
| Gender |  |  |  |
| Male | 8(61.5%) | 4(30.8%) | 0.116 |
| Female | 5(28.5%) | 9(69.2%) |  |
| ECOG PS |  |  |  |
| 0-1 | 13(100.0%) | 13 (100.0%) | 1.000 |
| 2 | 0(0.0%) | 0(0.0%) |  |
| Smoking status |  |  |  |
| Nonsmoker | 7(41.8%) | 13 (100.0%) | 0.015 |
| Smoker | 6(58.2%) | 0(0.0%) |  |
| Clinical staging |  |  |  |
| IIIB | 13 (100.0%) | 13 (100.0%) | 1.000 |
| IV | 0(0.0%) | 0(0.0%) |  |
| *EGFR* mutation status |  |  |  |
| 19 deletion | 8(61.5%) | 10(66.7%) | 0.673 |
| L858R mutation | 5(28.5%) | 3(33.3%) |  |
| others |  |  |  |

Abbreviations: Bev+CP, bevacizumab, carboplatin, and paclitaxel; GP: gemcitabine plus cisplatin; ECOG PS, Eastern Cooperative Oncology Group performance status; EGFR, epidermal growth factor receptor.

**Table S2. PFS of bevacizumab plus CP in clinical trials and our study**

| Studies | Patients | Study design | PFS or TTP (months) | OS(months) |
| --- | --- | --- | --- | --- |
| **ECOG 4599** | Western population | Bev+CP vs CP | 6.2 vs 4.5 (P<0.001) | 12.3 vs. 10.3 (P=0.030) |
| **SAiL** | Patients from 40 countries | Bev plus standard chemotherapy | 7.8 | 14.6 |
| **BEYOND** | Chinese patients | Bev+CP vs Pl+CP | 9.2 vs. 6.5 (P<0.001) | 24.3 vs. 11.7 (P=0.015) |
|  | Chinese patients with EGFR mutations | Bev+CP vs Pl+CP | 12.4 vs. 7.9 (P<0.050) | 24.3 vs. 27.5 (P>0.050) |
| **Our study** | Chinese patients with EGFR mutations | Bev+CP vs GP | 11.7 vs 4.7 (P=0.001) | 33.7 vs. 27.8 (P=0.293) |

Abbreviations: Bev+CP, bevacizumab plus carboplatin and paclitaxel; CP, carboplatin and paclitaxel; GP, gemcitabine and cisplatin; Pl, placebo; OS, overall survival; PFS, progression-free survival.
